# No association between ambient particulate matter exposure during pregnancy and stillbirth risk in the north of England, 1962–1992

**DOI:** 10.1016/j.envres.2009.10.003

**Published:** 2010-01

**Authors:** M.S. Pearce, S.V. Glinianaia, J. Rankin, S. Rushton, M. Charlton, L. Parker, T. Pless-Mulloli

**Affiliations:** a Institute of Health and Society, Newcastle University, Sir James Spence Institute, Royal Victoria Infirmary, Newcastle upon Tyne, NE1 4LP, UK; b Institute for Research in Environment and Sustainability, Newcastle University, UK; c National Centre for Geocomputation, National University of Ireland, Maynooth, Ireland; d Departments of Medicine and Pediatrics, Dalhousie University, Halifax, Nova Scotia, Canada

**Keywords:** Air pollution, Black smoke, Epidemiology, Particulate matter, Stillbirth

## Abstract

**Objectives:**

Research evidence suggests that exposure to ambient air pollutants can adversely affect the growth and development of the foetus and infant survival. Much less is known regarding the potential for an association between black smoke air pollution and stillbirth risk. This potential association was examined using data from the historical cohort UK Particulate Matter and Perinatal Events Research (PAMPER) study.

**Methods:**

Using data from paper-based neonatal records from the two major maternity hospitals in Newcastle upon Tyne (UK), a birth record database of all singletons born during 1961–1992 to mothers resident in the city was constructed. Weekly black smoke levels were obtained from routine data recorded at 20 air pollution monitoring stations over the study period. A two-stage statistical modelling strategy was used, incorporating temporally and spatially varying covariates to estimate black smoke exposure during each trimester and for the whole pregnancy period for each individual pregnancy. Conditional logistic regression models, with stratification on year of birth, were used to assess potential associations between black smoke exposures in pregnancy and stillbirth risk.

**Results:**

The PAMPER database consists of 90,537 births, between 1962 and 1992, with complete gestational age and residential address information, of which 812 were stillborn. There was no association between black smoke exposures in any trimester or across whole pregnancy and risk of stillbirth. Adjustment for potential confounders did not alter these results.

**Conclusions:**

While black smoke in pregnancy is likely to be related to other pregnancy outcomes, our findings do not suggest that black smoke air pollution exposure during pregnancy increases the risk of stillbirth.

## 1 Introduction

Evidence on the potential impact of air pollution on the health of adults and children has grown rapidly over the last two decades. It is now established that short-term and long-term increases in ambient air pollution are associated with increased mortality and morbidity in adults and children (Holgate et al., 1999; COMEAP, 1998; Katsouyanni et al., 1997; Brunekreef et al., 1995). Even at levels within contemporary air quality standards it is estimated that over 8000 deaths per year are being brought forward in the UK by exposure to particulate matter (COMEAP, 1998). Furthermore, in other parts of the world, coal combustion combined with traffic pollution remains a main source of high level particulate matter (Wang et al., 1997). Considerable consistency for many health effects exists across studies for particulate air pollution (measured as PM_10_, particulate matter with aerodynamic diameter of 10 μm or less, PM_2.5_ or previously as black smoke or total suspended particulates) (Schwartz et al., 1996; Dockery and Pope, 1994; Pope et al., 1995; Pope, 2000). The effects are more pronounced in susceptible population groups, such as the elderly or people with pre-existing cardiovascular or respiratory disease (Pope, 2000; Gouveia and Fletcher, 2000). Associations between long-term, or chronic, exposure to PM and an increase in total mortality, cardiovascular mortality and respiratory morbidity have also been reported (Pope et al., 1995; Pope, 2000).

A growing body of evidence now also suggests that exposure to ambient air pollutants, including particulates, can adversely affect the growth and development of the foetus (growth restriction, preterm birth, congenital anomalies) (Dejmek et al., 2000; Parker et al., 2005; Ritz et al., 2002; Leem et al., 2006) and infant survival (Woodruff et al., 1997; Lipfert et al., 2000; Bobak and Leon, 1999). A systematic review of the effect of PM exposure on foetal outcomes concluded that the currently available evidence was consistent with a small adverse effect of particulate air pollution on foetal growth and duration of pregnancy (Glinianaia et al., 2004).

Previous research into risk of stillbirth associated with particulate air pollution is limited. The three studies included in the systematic review reported little evidence of an association between exposure to particulate matter and stillbirth risk (Bobak, 1999; Sakai, 1984; Pereira et al., 1998). However, these studies were ecological or time-series in design with relatively crude measures of exposure. The systematic review concluded that the current evidence was insufficient to evaluate a potential association between particulate air pollution and stillbirth risk.

The aim of this study was to investigate further the potential association between ambient particulate matter and stillbirth risk, using individual-level information from the UK Particulate Matter and Perinatal Events Research (PAMPER) births database, an historical cohort study of all singleton births in Newcastle upon Tyne during 1961–1992 (Glinianaia et al., 2008).

## 2 Materials and methods

The PAMPER birth cohort consists of all singletons born during 1961–1992 to mothers resident in the city of Newcastle upon Tyne (current population approximately 260,000) in the northern region of England. The population structure of the northern region is characterised by a low percentage of ethnic minorities, of about 2% and a relatively stable population with low levels of both inward and outward migration (Office for National Statistics, 2002). A computer database of birth records was constructed using data from several sources. The main source was paper-based neonatal records from the two major maternity hospitals in Newcastle (the Princess Mary Maternity Hospital (PMMH), where neonatal records were available for whole study period, and Newcastle General Hospital (NGH), where records were available from May 1967 onwards). Neonatal records from both hospitals contained data on important maternal and foetal/infant characteristics including maternal name, home address at delivery, baby’s name, sex, date and time of birth and birth outcome (birth weight, gestational age, vital status, neonatal mortality and morbidity, major congenital anomalies). Data from ‘birth ledgers’, essentially a list of all births whether born in hospital or not, but also containing information on mother’s current surname and residential address, and the baby’s sex, date of birth, place of birth and vital status at birth, covering the period 1961–1973 were also included to establish the full denominator for the early period of the study. Additional birth records from the NGH stored in the Tyne & Wear Archives were accessed to complete some key variables, which were lacking in the birth ledgers. However, home births, except for the few where the mother was admitted to a maternity hospital shortly after birth, were only recorded on the birth ledger and so were without gestational age information. Therefore, home births were excluded from this particular study as it was not possible to assign an exposure estimate without gestational age information. Home births constituted about a third of all births in the early 1960s, about 10% in 1968 and was less than 1% by 1973. Further details of the construction of the PAMPER database are published elsewhere (Glinianaia et al., 2008).

The birth data were linked to information on stillbirths from the Office for National Statistics (ONS) for 1961–1992 to obtain data on cause of death. The linkage was based on date of birth, vital status (stillbirth), place of delivery and infant sex for 1961–1962. For 1963–1992 baby’s birth weight (where recorded), maternal age category and multiple birth indicator were also available as additional linkage variables. Among the total of 1248 eligible (resident within the study area and born in either NGH or PMMH, including home births for the 1960s) stillbirths provided by the ONS for the study period, 1222 (97.9%) were matched to the PAMPER database. For cross-validation we also linked PAMPER data to stillbirth data from the Northern Perinatal Mortality Survey (PMS) (available form 1981 onwards) (Northern Region Health Authority Coordinating Group, 1984). Causes of death obtained from the ONS were classified according to the International Classification of Diseases (ICD) revisions: revision 7 (covers 1958–67), revision 8 (1968–1978) and revision 9 (1979–2000). Causes of death for 1981–1992 were cross-checked with those obtained from the PMS and amended according to the PMS coding in an exceptional case of discrepancy.

For consistency across the study period we defined a stillbirth as the birth of a dead baby at 28 or more completed weeks of gestation (the legal cut-off in gestational age for stillbirth was changed to 24 weeks in October 1992 in England and Wales). Four stillbirths with unknown gestational age and birth weight less than 500 g were excluded.

All births were assigned unique spatial identifiers (postcodes and/or grid references). For births before 1970 (i.e. prior to the introduction of postcodes), the address at birth was assigned a postcode from the 1991 postcode book and converted to a grid reference (equivalent to *X*–*Y* co-ordinates) or, where a complete postcode could not be identified, a grid reference directly. Townsend Deprivation Score (TDS), an area-based measure of socio-economic status incorporating the proportion of home ownership, car ownership, unemployment and overcrowding, was calculated at the enumeration district level using data from the 1971, 1981 and 1991 UK Census Surveys.

### 2.1 Exposure assessment

Black smoke is a historic measure of airborne particulate matter. Daily black smoke levels, with black smoke approximately equivalent to PM_4_, were obtained from routine data recorded at 20 air pollution monitoring stations within Newcastle upon Tyne’s city boundary between October 1961 and December 1992 and available from the UK Air Quality Archive. The city boundary used was based on the postcodes NE1-NE7 and part of NE15, excluding Throckley, with the River Tyne forming the Southern boundary. An exact map of the study area is available in a previous publication from the study (Glinianaia et al., 2008). Historical records were used to identify industrial land usage and numbers of residential and industrial chimneys. Over the whole study period, the number of monitors active in the study area during any given week varied between three and ten. Black smoke levels for each individual birth were estimated using a combination of air pollution data, date of birth, estimated date of conception (based on date of last menstrual period) and the mother’s residential postcode which identified the location at which black smoke levels were to be estimated.

The modelling process to estimate individual exposure estimates is described in detail elsewhere (Fanshawe et al., 2008). Briefly, a two-stage modelling strategy was employed. First, a seasonally varying temporal trend in black smoke exposures was estimated using a dynamic linear model. Secondly, the remaining spatio-temporal variation was accounted for using temporal and/or spatial covariates (number of chimneys within 500 m of monitor, distance of monitor to nearest industry, type of land-use and implementation of the Clean Air Act). The residual spatio-temporal correlation remaining after this process was negligible. The two-stage exposure model used for individual exposure estimation explained 84% of the variation in black smoke levels at the locations of the monitoring stations. Mean weekly exposures were estimated for each birth averaged across each trimester of pregnancy and also across the whole pregnancy period. As air pollution data were only available from October 1961, only births with complete exposure estimates for the whole of pregnancy were included.

### 2.2 Statistical analysis

Potential associations between estimates of black smoke and risk of stillbirth were investigated using conditional logistic regression, with stratification on year of birth to account for the dramatic decrease in stillbirth rates over the study period. An analysis by time period (1962–1972, 1973–1982 and 1983–1992) is also presented. Odds ratios (ORs) per 10 μg/m^3^ and corresponding 95% confidence intervals (95% CIs) are reported. Although variables are presented categorically in descriptive tables, all conditional logistic regression analyses used continuous, or for parity, ordinal, data for all variables other than infant sex. A further analysis limiting cases of stillbirth to those due to antepartum hypoxia, which reflect intrauterine growth retardation, was also done.

The statistical software package Stata, version 10, (StataCorp, College Station, TX) was used for all statistical analyses.

### 2.3 Ethics

The study received a favourable ethical opinion from the Sunderland Local Research Ethics Committee.

## 3 Results

The PAMPER database contains details of 109,080 births. Of the 90,537 births with complete gestational age information and black smoke exposure estimates, 812 were stillborn (430 male, 379 female and three of unknown sex). The distribution of births included in this study by time period, sex parity and maternal age is shown in Table 1. The distribution of mean weekly black smoke estimates for each trimester of pregnancy and the whole pregnancy period, by time period, are shown in Table 2. There was a dramatic decrease in exposures between the time periods, with a correlation between whole pregnancy black smoke exposures and year of birth of −0.84. Missing data for parity and maternal age were more common in earlier years and associated with higher estimated black smoke exposures. The median gestational age for stillbirths was 36 weeks (inter-quartile range 32–39), in live births the median was 40 weeks (inter-quartile range 39–40).

There were no significant associations between stillbirth risk and any of the black smoke exposure estimates during pregnancy, either for the whole study period or in any of the time periods considered (Table 3). Adjusting for infant sex, maternal age, parity and Townsend deprivation score made very little difference to the results (Table 3). Results (not shown) were very similar when stillbirths were restricted to those due to antepartum hypoxia (*n*=563).

## 4 Discussion

A growing body of evidence suggests that exposure to ambient air pollutants, including particulates, can adversely affect the growth and development of the foetus (including growth restriction, preterm birth and congenital anomalies) (Dejmek et al., 2000; Parker et al., 2005; Ritz et al., 2002; Leem et al., 2006) and infant survival (Woodruff et al., 1997; Lipfert et al., 2000; Bobak and Leon, 1999). However, the results of this study of over 800 stillbirths are consistent with previous studies suggesting no association between stillbirth risk and particulate matter exposure during pregnancy (Bobak, 1999; Sakai, 1984; Pereira et al., 1998).

In contrast to the previous studies which have been ecological or time-series in design with relatively crude measures of exposure, this study used individual-level exposure estimates on a large unselected birth cohort. Completeness of the PAMPER database both for the number of births and collected information for each birth is one of the evident strengths (Glinianaia et al., 2008). The study area covers a clearly defined conurbation with high quality records of land-use, including historical records of industrial usage and numbers of residential and industrial chimneys. We were also able to allow for temperature and season in the exposure estimation process, taking account of the seasonal variation in black smoke estimates and the decrease in black smoke air pollution over the study period. Although from a small geographical region, due to the long time period covered, the study included large variations in black smoke levels.

The black smoke recordings used in this study were collected routinely over the study period, although not all monitors were in place throughout the study period, creating likely geographical differences in uncertainties surrounding our estimated exposures. In addition, monitoring procedures reflected best practise at the time and are thus the best estimates available, although their accuracy would have varied over time.

Residential mobility during pregnancy may be associated with exposure misclassification and therefore may introduce bias. It has been reported for the United States, Canada and Australia that 25–33%, 12% and 19% of the population move during pregnancy, respectively (Fell et al., 2004; Khoury et al., 1988; Raynes-Greenow et al., 2008; Shaw and Malcoe, 1992; Zender et al., 2001). Whilst we do not have the residential mobility data for the study cohort we have indirect evidence of population stability for children and older women (Pless-Mulloli et al., 1998; Edwards et al., 2006; Pless-Mulloli et al., 2000). For the period 1985–2003 we have evidence that only 9% of women moved during pregnancy with a median moving distance of 1.4 km (Hodgson et al., 2008).

The accuracy of gestational age estimates is important for epidemiologic studies of pregnancy outcomes. Different methods for gestational age assessment (based on the last normal menstrual period (LMP) or early ultrasound measurements) throughout the study period may introduce bias in gestational age estimation over time. However, while creating our birth record database, we made the recording of gestational age as objective and accurate as possible by accepting gestational age calculated from the recorded estimated date of delivery (EDD) (i.e. LMP based) for the majority of births rather than by entering gestational age recorded in the neonatal notes or birth records. In this study the ultrasound age estimate has been used since the early 1980s only for pregnancies with uncertain date of LMP or if there was a significant discrepancy between the two estimates, therefore it should not bias gestational age estimates over time (Glinianaia et al., 2009). Further, as the live and stillbirths in this study were effectively matched on year of birth, any difference in the quality of records over time would be unlikely to introduce a bias.

As the legal cut-off in gestational age for stillbirth was changed to 24 weeks in October 1992 in England and Wales (Still-Birth (Definition) Act 1992, 1993), only stillbirths at 28 or more completed weeks of gestation were included for consistency across the whole study period. As the final birth in this study was in December 1992, this has only resulted in a small number of stillbirths being excluded from the study due to being delivered at a gestational age between 24 and 28 weeks. However, as with any cut-off for gestational age, the use of a 28-week cut-off, as it applied for the vast majority of the study period does mean that data are not available for any earlier foetal losses. The potential effect of this definition on this and other similar studies is uncertain and would require more detailed data on foetal losses throughout pregnancy rather than during the period for which routine data collection takes place in most countries.

Given the strong inverse correlation between black smoke exposure during pregnancy and year of birth (*r*=−0.84) and the substantial decline in stillbirth rates over the study period, year of birth is likely to confound any association between stillbirth risk and black smoke. Therefore we chose to use a conditional model, stratified by year of birth. This effectively matches stillbirths to live births by year of birth, though maintains the entire dataset for analysis and utilises the entire exposure range across a year, while adjusting for the known decrease in stillbirth rates over time. We also analysed the data in three different time periods to address whether associations may only be evident in certain time periods, for example due to much higher exposures in the earlier years of the study, or due to the changing composition of black smoke air pollution over the study period. No significant associations were seen in any of these analyses.

While black smoke in pregnancy may be adversely related to other pregnancy outcomes, our findings do not suggest that black smoke air pollution exposure during pregnancy increases the risk of stillbirth.

## Figures and Tables

**Table:** Live births and stillbirths by time period, parity, sex and maternal age.

	**Live births**	**Stillbirths**	**Stillbirth rate per 1000 total births**	**Odds Ratio**^b^**(95% CI)**
**Year of birth**				
1962–1972	28136	378	13.3	1.00
1973–1982	28733	243	8.4	0.63 (0.54–0.74)
1983–1992	32855	188	5.7	0.43 (0.36–0.51)

**Parity**^a^				
0	39327	347	8.7	1.00
1	27043	179	6.6	0.79 (0.66–0.94)
2	12733	120	9.3	1.05 (0.85–1.30)
3	5209	64	12.1	1.30 (0.99–1.70)
4+	5312	99	18.3	1.60 (1.27–2.02)

**Sex**^a^				
Male	46317	430	9.2	1.00
Female	43407	379	8.7	0.94 (0.82–1.08)

**Maternal age**				
<20 years	11432	114	9.9	1.00
20–24	29142	225	7.7	0.75 (0.60–0.95)
25–29	27568	211	7.6	0.78 (0.62–0.98)
30–34	14505	146	10.0	1.00 (0.78–1.28)
35+	6342	112	17.4	1.52 (1.17–1.99)

a 4 births of unknown sex, 104 of unknown parity and 740 of unknown maternal age.

b Unadjusted odds ratios from conditional logistic regression model, except for year of birth where unstratified odds ratios are presented.

**Table 2:** Estimated mean weekly black smoke exposures (μg/m^3^) by time period and trimester of pregnancy and, for whole pregnancy, by time period and parity, sex and maternal age.

	**Year of birth**		
	**1962–1972 median (IQR)**	**1973–1982 median (IQR)**	**1983–1992 median (IQR)**
Trimester 1	136 (88–214)	37 (23–59)	15 (11–19)
Trimester 2	137 (88–209)	36 (23–58)	15 (11–19)
Trimester 3	132 (86–204)	34 (22–54)	14 (10–19)
Whole pregnancy	147 (110–197)	37 (22–54)	15 (12–19)

**Parity**			
0	146 (109–192)	38 (27–55)	15 (12–18)
1	137 (102–182)	37 (26–53)	15 (12–19)
2	146 (107–198)	37 (26–53)	15 (12–19)
3	150 (113–202)	37 (26–60)	16 (12–19)
4+	174 (133–227)	40 (29–63)	15 (12–18)

**Sex**			
Male	147 (110–196)	37 (27–55)	15 (12–18)
Female	148 (110–198)	37 (27–54)	15 (12–19)

**Maternal age**			
<20 years	147 (112–192)	41 (29–60)	16 (13–19)
20–24	148 (111–195)	39 (28–57)	16 (13–19)
25–29	143 (104–195)	36 (26–52)	15 (12–18)
30–34	147 (109–201)	34 (24–48)	14 (11–18)
35+	156 (120–207)	36 (25–55)	14 (11–18)

**Live births**	147 (110–197)	37 (27–54)	15 (12–19)
**Stillbirths**	167 (121–223)	39 (26–57)	16 (12–19)

IQR—Inter-Quartile Range

**Table 3:** Odds ratios per 10 (μg/m^3^) relating average weekly black smoke exposure and stillbirth.

	***Unadjusted***	***Fully adjusted***^a^
	**OR (95% CI)**	**OR (95% CI)**
**Trimester 1**		
Whole study period	1.001 (0.992–1.013)	1.001 (0.990–1.012)
1962–1972	1.002 (0.992–1.013)	1.001 (0.990–1.012)
1973–1982	1.012 (0.958–1.068)	1.010 (0.955–1.067)
1983–1992	0.840 (0.638–1.106)	0.782 (0.588–1.039)

**Trimester 2**		
Whole study period	1.005 (0.994–1.016)	1.004 (0.993–1.015)
1962–1972	1.004 (0.993–1.015)	1.003 (0.992–1.014)
1973–1982	1.023 (0.970–1.078)	1.019 (0.965–1.076)
1983–1992	0.974 (0.745–1.274)	0.937 (0.711–1.234)

**Trimester 3**		
Whole study period	1.005 (0.995–1.016)	1.004 (0.993–1.015)
1962–1972	1.006 (0.995–1.017)	1.006 (0.994–1.017)
1973–1982	0.988 (0.933–1.046)	0.981 (0.925–1.039)
1983–1992	0.930 (0.707–1.222)	0.872 (0.657–1.159)

**Whole pregnancy**		
Whole study period	1.012 (0.995–1.003)	1.010 (0.991–1.028)
1962–1972	1.012 (0.994–1.030)	1.010 (0.991–1.030)
1973–1982	1.033 (0.948–1.127)	1.024 (0.936–1.121)
1983–1992	0.853 (0.550–1.322)	0.721 (0.453–1.147)

a Adjusted for parity, maternal age, sex and Townsend deprivation score.
